# PP57 Proposal For Adaptation Of The Pharmaceutical Innovativeness Index Based On Lenacapavir

**DOI:** 10.1017/S0266462325102766

**Published:** 2025-12-29

**Authors:** Alex Martins, Ludmila Gargano, Francisco Acurcio, Juliana Alvares-Teodoro, Augusto Guerra

## Abstract

**Introduction:**

Treatment of HIV-1 infection is based on antiretroviral therapy. A pharmaceutical innovativeness index (PII) was proposed as a tool to evaluate the degree of innovativeness of technologies, initially for cancer treatment. This study aimed to adapt the PII for assessing therapies used in the treatment of people living with multidrug-resistant HIV-1 (MDR-HIV), based on an assessment of lenacapavir.

**Methods:**

The PII is structured into four domains: unmet therapeutic need (UTN), added therapeutic value (ATV), study design (SD), and methodological quality (MQ). The second domain required adaptation to better fit the outcomes typically evaluated in pivotal studies of antiretrovirals for the treatment of MDR-HIV patients. To adapt the PII for antiretroviral therapy innovations, we assessed the innovativeness of lenacapavir as a case study, based on the phase two/three study comparing it to fostemsavir trometamol and its pivotal phase three study.

**Results:**

In this adaptation, virological response rate ratio replaced relative risk of mortality in the ATV domain. The proposed scale suggested that a ratio less than one corresponded to “absent” ATV; a ratio between 1.01 and 1.24 to “poor” ATV; between 1.25 and 1.99 to “moderate” ATV; and a ratio of at least two to “maximum” ATV. Lenacapavir was classified as “moderate” for both UTN and ATV domains, as “inadequate” for study design (single-arm trial), and as low risk of bias in the MQ domain. Thus, an innovativeness score of 50 was assigned to lenacapavir.

**Conclusions:**

This preliminary case study adaptation provided an important contribution to evaluating the degree of innovativeness of antiretrovirals used in the treatment of people living with MDR-HIV. Maintaining the weights assigned to each domain proposed by the PII, led to an innovation score of 50 for lenacapavir.

